# The Interaction of pT73-Rab10 with Myosin Va, but Not Myosin Vb, Is Regulated Though a Site in the Globular Tail Domain

**DOI:** 10.3390/cells14151140

**Published:** 2025-07-24

**Authors:** Lynne A. Lapierre, Elizabeth H. Manning, Kyra S. Thomas, Catherine Caldwell, James R. Goldenring

**Affiliations:** 1 Department of Surgery, Vanderbilt University Medical Center, Nashville, TN 37232, USA; 2 Epithelial Biology Center, Vanderbilt University School of Medicine, MRB IV 10435G, 2213 Garland Avenue, Nashville, TN 37232, USA; 3 Nashville VA Medical Center, Nashville, TN 37212, USA; 4 Department of Cell and Developmental Biology, Vanderbilt University School of Medicine, Nashville, TN 37232, USA

**Keywords:** Rab10, Myosin VA, Myosin VB, Rab11a, LRRK2

## Abstract

The phosphorylation of Rab10 (pT73-Rab10) by LRRK2 promotes the establishment of epithelial cell polarity by controlling the trafficking to the primary cilia membrane of cilia-resident proteins and signaling proteins. Previous studies have identified a site in the globular tail domain of MYO5A that specifically binds to only the phosphorylated form of Rab10. In this work, we have demonstrated that pT73-Rab10 does not associate with the globular tail of MYO5B. We have mapped the putative binding site to a required three amino acids (MEN, 1473–1475) in the MYO5A globular tail domain that are not found in the MYO5B globular tail. Substitution of the MEN amino acid sequence found in MYO5A into the paralogous position in the MYO5B globular tail conferred the ability to associate with pT73-Rab10. The results demonstrate that the interactors with MYO5A and MYO5B are not completely overlapping and that the interaction of pT73-Rab10 is specific to the MYO5A globular tail domain.

## 1. Introduction

Mutations in Leucine-Rich Repeat Kinase 2 (LRRK2) constitute the predominant genetic cause of Parkinson’s disease, with the most common mutations leading to a constituently active LRRK2 [1]. Utilizing a phospho-proteomic screen to identify LRRK2 substrates, the Mann lab identified 14 Rab proteins phosphorylated in the switch II domain by LRRK2 [2,3]. Of these 14 Rabs, 10 Rabs, including Rab3a/b/c/d, Rab5a/b/c, Rab8a/b, Rab10, Rab12, Rab29, Rab35, and Rab43, were phosphorylated in the switch II domain at endogenous levels and exhibited a significant decrease in phosphorylation when cells were treated with the LRRK2 inhibitor MLi-2 [2].

LRRK2 phosphorylates Rab10 on threonine 73 (pT73-Rab10). Rab10 and pT73-Rab10 have been implicated in multiple cellular functions, including basolateral trafficking, ER dynamics and morphology, mitophagy, and lysosomal homeostasis [4,5,6]. Recently, studies have focused on Rab10/pT73-Rab10 in centrosome stability and primary cilia growth. In 2010, the Dunn lab located Rab10 in the primary cilia, where Rab10 colocalized with components of the exocyst [7]. The overexpression of LRRK2 in HeLa cells led to high levels of pT72-Rab8 and pT73-Rab10, resulting in the accumulation of the phosphorylated Rabs at the centrosome and a reduction in centrosome cohesion [8,9]. These effects on centrosome cohesion and primary cilia growth have been linked to pT73-Rab10 interactions with RILPL (Rab Interacting Lysosomal Protein Like) 1 and 2 [3,8,9].

We had previously shown that Rab10 interacts with Myosin V family members though the alternatively spliced exon D [10]. The Pfeffer lab recently reported a high affinity binding site within the globular tail domain (GTD; aa-1421–1880) of Myosin Va (MYO5A) that is specific for the phosphorylated form of Rab10 [11]. In the present work, we have found that pT73-Rab10 does not associate with the globular tail of MYO5B. We have mapped the pT73-Rab10 putative binding site to amino acids 1473–1475 within the globular tail of MYO5A. Interestingly, this stretch of amino acids is not contained in the globular tail of MYO5B. To date, the Rab proteins identified as interactors with one member of the Myosin V family were also found to interact with the other Myosin V family members. While Rab10 can interact with both MYO5A and MYO5B through the alternatively spliced Exon D [10], the phosphorylated form of Rab10 associates only with the globular tail of MYO5A and not with the globular tail of MYO5B. All previous work on pT73-Rab10 has been achieved by overexpressing LRRK2 or pathogenic R441G-LRRK2. In this investigation, we have utilized A549 lung cells, which manifest detectable levels of endogenous pT73-Rab10. By overexpressing the wild type and mutated forms of the MYO5A and MYO5B globular tails, as opposed to overexpressing LRRK2, we have sought to define the requirements for the pT73-Rab10 association with MYO5A.

## 2. Materials and Methods

### 2.1. Vector Construction

DNA sequences for the globular tail domains (GTDs) of mouse MYO5A (1438–1880), MYO5A (1438–1880; MYO5A-GT-S) and MYO5A (1497–1880) and human MYO5B (1408–1848) were cloned into pEGFP-C2 (Clontech). MYO5B (902–1848) was cloned into pCherry-C2. Site-directed mutagenesis of the GTD sequences was performed (see Table 1 for the sequences) utilizing the QuickChange Lightening Multi Site-Directed Mutagenesis Kit (Cat #: 210523; Agilent, Santa Clara, CA, USA). All mutagenized and cloned sequences were confirmed using Sanger sequencing (GenHunter, Nashville, TN, USA) and whole plasmid sequencing was performed by Plasmidsaurus using Oxford Nanopore Technology with a custom analysis and annotation.

### 2.2. Cell Culture and Transfections

The A549 human epithelial lung cell line [12] (ATCC, Manassas, VA, USA) and Madin–Darby Canine Kidney (MDCK) cells were grown in D-MEM supplemented with 10% FBS. Cells were plated onto coverslips for immunofluorescence staining or 6-well plates for western blotting and transfected with the indicated vectors using PolyJet (SignaGen Labs, Frederick, MD, USA, cat. #: SL100688). Cells were analyzed at confluence. To inhibit the LRRK2 kinase, cells were treated for 18–24 h with 200 nM MLi-2 (Tocris Biosciences, Minneapolis, MN, USA, cat. #: 5756).

### 2.3. Immunofluorescence and Colocalization

**Figure 1:**
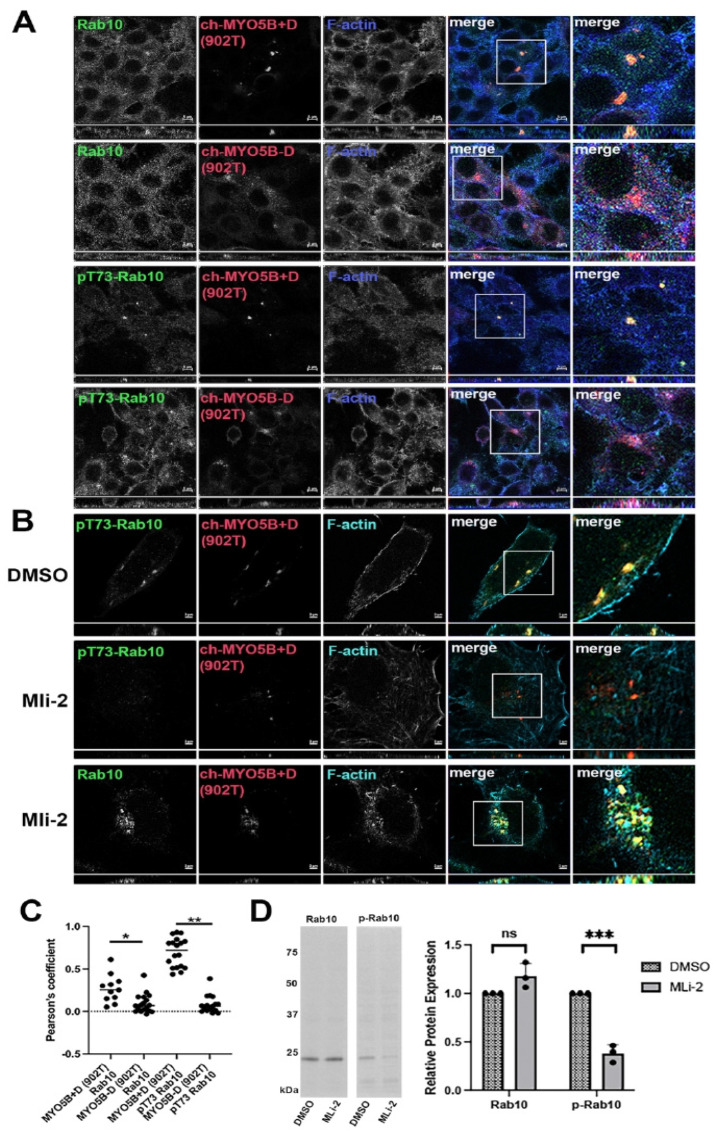
pT73-Rab10 and Rab10 associate with MYO5B+D but not with MYO5B without exon D. The A549 cell lines transfected with pCherry-MYO5B 902 tail (902T) with (+) and without (Δ) exon D were stained for either Rab10 or pT73-Rab10 and phalloidin. X-Y images are shown with *z*-axis projections below each. Higher resolution inset images are shown to the right. (**A**) Rab10 and pT73-Rab10 both bound to Ch-MYO5B-902T plus exon D but not minus exon D. (**B**) The Ch-MYO5B 902 tail + D cell line was treated for 18 h with DMSO or MLi-2 then stained for Rab10 or pT73-Rab10. MLi-2 blocked the phosphorylation of Rab10 but did not block Rab10 from binding to the Ch-MYO5B+D(902T). (**C**) Quantitation of the colocalization in A. * *p* = 0.0035, ** *p* < 0.001. (**D**) Rab10 and pT73-Rab10 expression were measured in protein lysates from A549 cells after 24 h of treatment with 200 nM MLi-2 or DMSO by western blotting. The western blot on the left shows a representative blot for Rab10 and p73-Rab10 (p-Rab10). Across three replicate experiments, the mean intensity of the Rab10 band in the MLi-2-treated cells was 1.18 ± 0.0752 standard error and an unpaired *t*-test showed a non-significant *p*-value of 0.0762 between MLi-2- and DMSO-treated cells. The mean intensity of the p-Rab10 band in the MLi-2-treated cells was 0.381 ± 0.051 standard error and an unpaired *t*-test showed a significant *** *p*-value of 0.0003 between MLi-2- and DMSO-treated cells. Bar = 2 μm.

**Figure 2:**
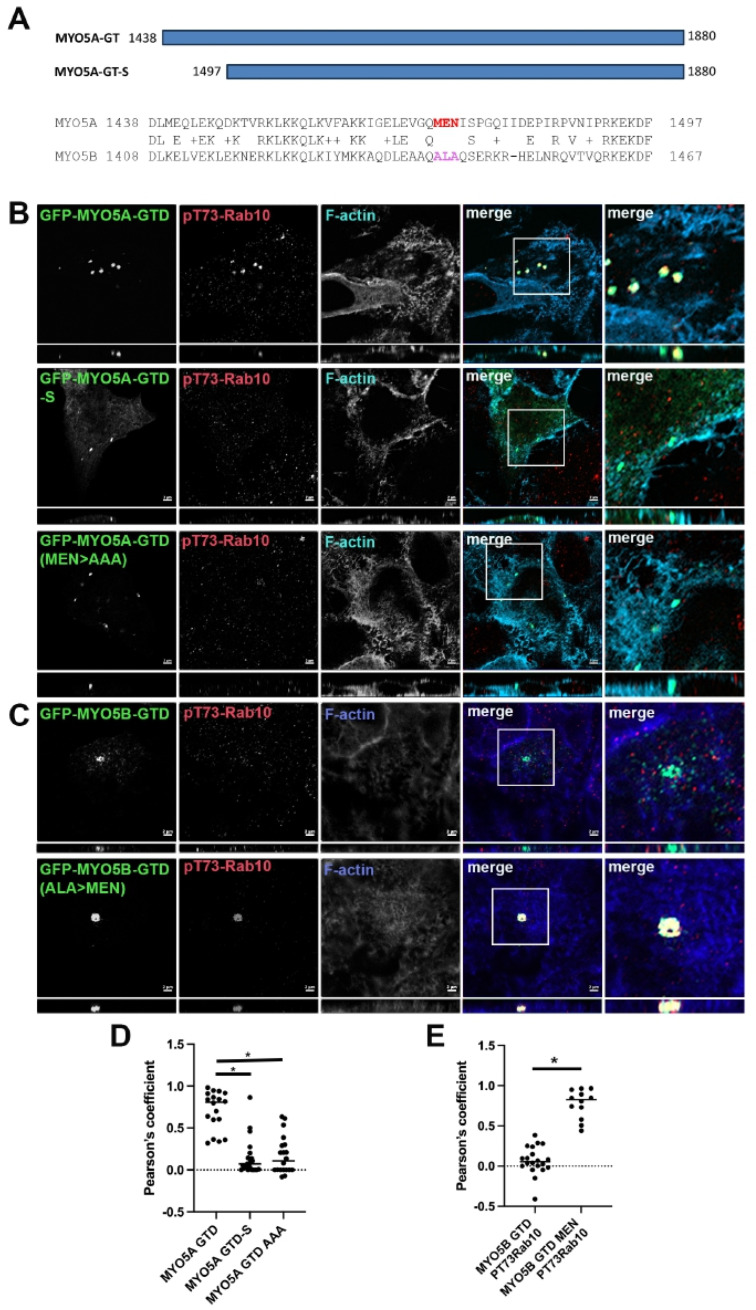
Truncation of the GTD or mutation of amino acids MEN to AAA in the GTD of MYO5A abolished pT73-Rab10 binding. (**A**) Schematic drawing of the MYO5A globular tail domain (MYO5A-GTD) and the shortened globular tail domain (GTD-S). The alignment of MYO5A and MYO5B shows the critical region with the MEN sequence in MYO5A and the paralogous ALA sequence in MYO5B. (**B**) A549 cells were transiently transfected with GFP-MYO5A GTD, GFP-MYO5A GTD-S or with the GFP-MYO5A GTD with the mutation of amino acids MEN to AAA (green). X-Y images are shown with *z*-axis projections below each. Higher resolution inset images are shown on the right. (**C**) A549 cells were transiently transfected with GFP-MYO5B GTD or with the GFP-MYO5B GTD with the mutation of ALA to MEN (green). The next day, the cells were fixed with 4% paraformaldehyde and stained for pT73-Rab10 (red) and phalloidin (blue). Bar = 2 μm. (**D**) Quantitation of the colocalization in A, * *p* < 0.001. (**E**) Quantitation of the colocalization in B, * *p* < 0.001.

Transfected cells were fixed with MeOH for 10 min at −20 °C or 4% paraformaldehyde for 20 min at room temperature, then blocked, and extracted in 10% normal donkey sera (Jackson ImmunoResearch, West Grove, PA, USA, cat. #: 017-000-121), 0.3% Triton X-100, and PBS for 30 min at RT. For the primary antibodies used, see Table 2. All secondary antibodies used were from Jackson ImmunoResearch. Stained cells were mounted with ProLong Gold (Invitrogen, Carlsbad, CA, USA, cat. #: P10144). Cells were imaged on a Zeiss LSM 980 with an Airyscan 2 confocal microscope using an 63x/1.40 Plan-Apochromat oil immersion lens and 4X zoom in Airyscan mode. Images were processed utilizing Zeiss ZEN Blue version 2.3 software, and the figures were assembled in Adobe Photoshop. Colocalization was quantified in individual GFP- or Cherry-expressing cells with Imaris version 10.1 software (Oxford Instruments). The 3D ROIs were manually created by drawing an ROI around the expressing cell on the slice corresponding to the widest point of the cell. This ROI was then copied and pasted to the slices, defining the top and bottom of the cell using the Create Surface function. A mask of just the ROI was created with the Mask Channel option. The GFP or Cherry channel was chosen then duplicated. Outside voxels were set to zero and the new mask channel was saved and used for the colocalization analysis. The new masked ROI was used to analyze the colocalization of the GFP or Cherry fluorescence with the stained fluorescence utilizing the calculate threshold function. Quantitation was performed in at least 3 fields containing 1–3 cells per field from 3 separate experiments. The calculated data were exported into Excel then imported into Prism to create Pearson’s graphs. Differences in Pearson’s coefficients were evaluated with a one-way ANOVA with a post hoc evaluation of significant means with Tukey’s test (Figure 1C and Figure 2D) or with an unpaired Student’s *t*-test (Figure 2E). The Zeiss 980 microscope and Imaris version 10.1 software are maintained in the Vanderbilt Cell Imaging Shared Resource.

### 2.4. Western Blot Analysis

A549 cells were grown on 6-well plates to ~70% confluence, treated for 24 h with 200 nM MLi-2 or DMSO, then washed with ice cold TBS, scraped, pelleted, and resuspended in 100 μL of 65 °C 2% SDS/10 mM EDTA/60 mM Tris and sonicated twice for 15 s. After the addition of Laemmli sample buffer, lysates were run twice on 4–12% polyacrylamide gels, then transferred onto an Odyssey nitrocellulose membrane (LiCor, Lincoln, Nebraska, cat #: 926-31092) for 1 h at 75 V. Membranes were blocked with 3% BSA/TBS (for p-Rab10) or Odyssey TBS Intercept blocking buffer (LiCor, cat. #: 927-60001 for Rab10), probed with rabbit anti-p-Rab10 or Rab10 antibodies overnight (see the Supplemental Table), and then probed with an Odyssey anti-rabbit 800 nm secondary antibody (LiCor, cat. #: 926-32213) for 1 h at room temperature. Blots were imaged using a LiCor Odyssey Fc. Membranes were then stained with Ponceau S (Sigma, St. Louis, MO, USA, cat. #: P7170) to visualize the total protein, and colorimetric images were taken with an Amersham Imager 680 (Marlborough, MA, USA). The densitometric analysis of western blot bands from three experiments was performed using ImageJ.JS (NIH). Protein levels were normalized to each lane’s corresponding Ponceau S signal. All data are presented as the means ± standard errors of the means (SEMs), and statistically significant differences were computed in GraphPad Prism 10.2.0 (Graph Software) using an unpaired *t*-test with the significance level set at *p* < 0.05.

## 3. Results

Evaluation of the association of phosphorylated Rab10 with MYO5B. We previously reported that Rab10 only interacted with the family of Myosin V motor proteins through the alternately spliced exon D. Recent work has shown that Rab10 is phosphorylated on threonine 73 (pT73Rab10) [2] by Leucine-Rich Repeat Kinase 2 (LRRK2). In A549 lung cells, antibodies against both Rab10 and pT73-Rab10 stained punctate vesicular structures in non-transfected cells (Figure 1). To investigate the association of pT73-Rab10 with MYO5A and MYYO5B, we utilized the overexpression of motorless tail constructs that have previously elicited the collapse of the Rab11a-containing vesicular recycling system [10,13]. Similar to unphosphorylated Rab10, the phosphorylated form of Rab10 (pT73-Rab10) only associated with the form of MYO5B C-terminal tail, aa 902–1849, containing Exon D (MYO5B 902T+D) (Figure 1A,C). We next tested if phosphorylation of Rab10 is necessary for the association with MYO5B 902T+D. Cells were treated for 24 h with either DMSO or the LRRK2 inhibitor MLi-2, and stained for pT73-Rab10 or total Rab10. Treatment with MLi-2 abolished staining for pT73-Rab10 by immunofluorescence (Figure 1B) and by western blotting (Figure 1D) but did not alter the interaction of non-phosphorylated Rab10 with MYO5B 902T+D (Figure 1B).

**Figure 3:**
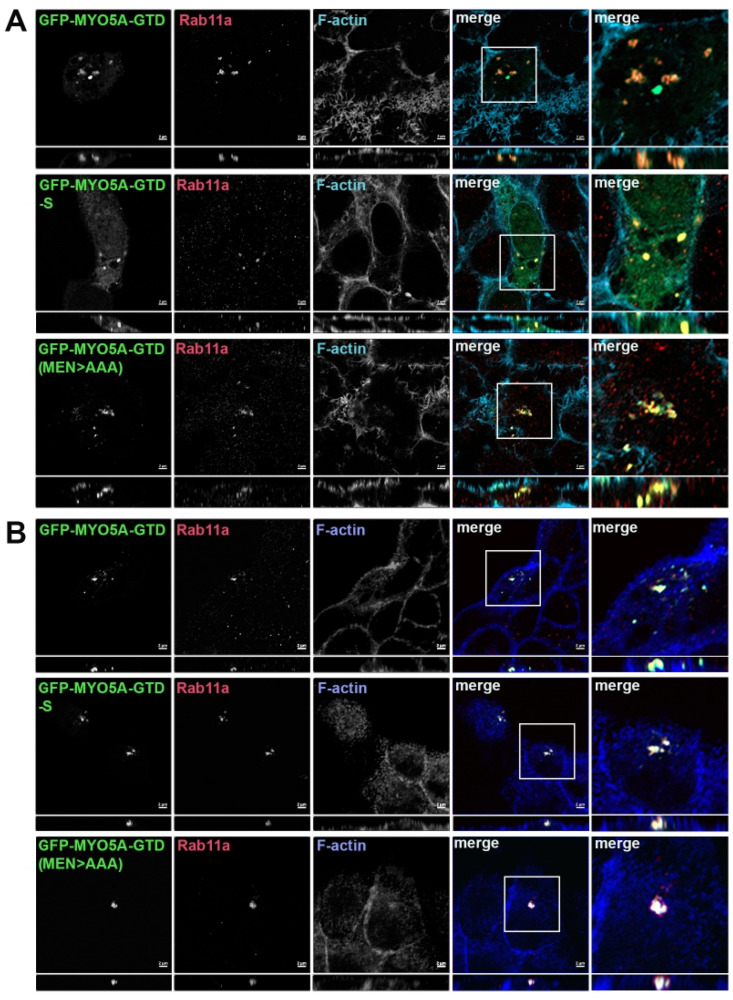
Truncation of the MYO5A GTD or mutation of MEN to AAA in the GTD of MYO5A did not alter the Rab11a association with MYO5A. A549 cells (**A**) or MDCK cells (**B**) were transiently transfected with GFP-MYO5A GTD, GFP-MYO5A GTD-S or with GFP-MYO5A GTD MEN > AAA mutation (green). The next day, the cells were fixed with 4% paraformaldehyde and stained for Rab11a (red) and phalloidin (blue). X-Y images are shown with *z*-axis projections below each. Higher resolution inset images are shown on the right. Bar = 2 μm.

Mapping the association of phosphorylated Rab10 with MYO5A. The Pfeffer lab identified a second binding site for pT73-Rab10 and only the phosphorylated form of Rab10 associated with the globular tail domain (GTD) of MYO5A (MYO5A-GTD) [11]. We first sought to define more precisely the region of the MYO5A-GTD responsible for binding pT73-Rab10 (Figure 2A). As previously reported, we did observe that pT73-Rab10 colocalized with MYO5A-GTD (Figure 2B, row 1). While the GTDs in MYO5A and MYO5B are very similar, there is a stretch on the N-terminus of the globular tail that does differ between the two MYO5 members (Figure 2A). Shortening the MYO5A-GTD to 1497–1880 (MYO5A-GTD-S) removed this stretch of heterogeneity and resulted in the loss of colocalization of pT73-Rab10 with MYO5A-GTD (Figure 2B, row 2), but not with Rab11a (Figure 3). Within this stretch of heterogeneity, there is a sequence of three charged amino acids that differ significantly between the two MYO5s, MEN in MYO5A and ALA in MYO5B (Figure 2A). Mutation of MEN to AAA in the globular tail domain of MYO5A (MYO5A-GTD(MEN > AAA)) disrupted the ability of pT73-Rab10 to colocalize with the mutated MYO5A-GTD (Figure 2B, row 3).

**Figure 4:**
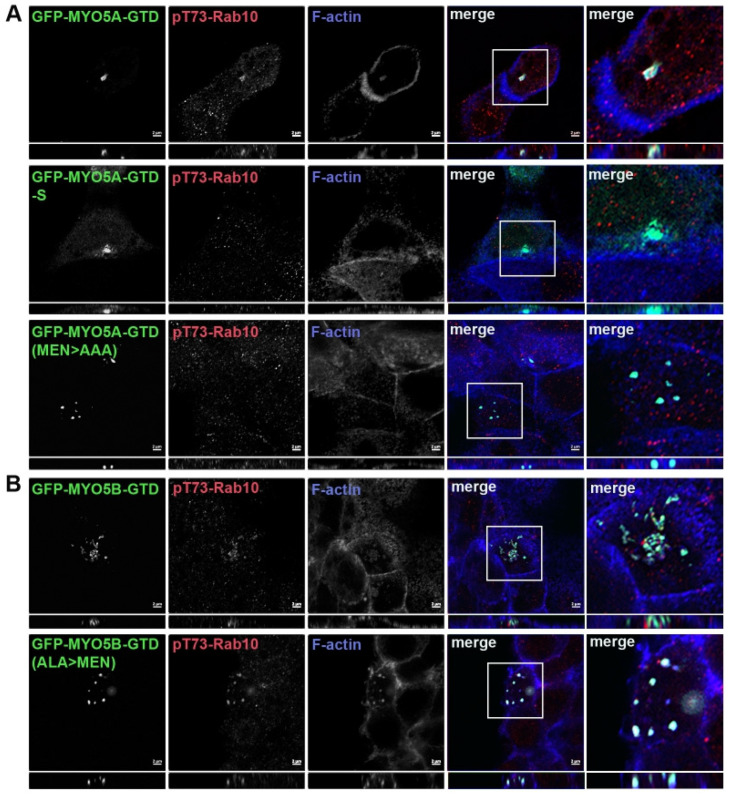
Truncation or mutation of the amino acids MEN to AAA in the GTD of MYO5A abolished the association with pT73-Rab10. (**A**) MDCK cells were transiently transfected with GFP-MYO5A GTD, GFP-MYO5A GTD-S or GFP-MYO5A GTD MEN > AAA mutation (green). (**B**) MDCK cells were transiently transfected with GFP-MYO5B GTD or GFP-MYO5A GTD ALA > MEN mutation (green). The next day, the cells were fixed with 4% paraformaldehyde and stained for pT73-Rab10 (red) and phalloidin (blue). X-Y images are shown with *z*-axis projections below each. Higher resolution inset images are shown on the right. Bar = 2 μm.

Association of phosphorylated Rab10 with the MYO5B globular tail. We tested MYO5B-GTD to ascertain if pT73-Rab10 would associate with just a GTD construct. No colocalization of pT73-Rab10 was observed with the MYO5B-GTD (Figure 2C, row 1). Mutation of the ALA sequence to MEN in the MYO5B-GTD (MYO5B-GTD(ALA > MEN)) resulted in pT73-Rab10 colocalization with the mutated MYO5B-GTD (Figure 2C, row 2). Rab11a colocalized with all forms of the MYO5A-GTD (Figure 3A), indicating that the truncation and the mutations of the MYO5A did not globally disrupt the GTD structure. Similar results were seen in MDCK cells (Figure 3B and Figure 4). Overall, these findings demonstrate that pT73-Rab10 interacts at a specific sequence in MYO5A that is absent in MYO5B.

## 4. Discussion

The most common cause of familial Parkinson’s disease is linked to mutations in the leucine rich-repeat kinase 2 (LRRK2) protein [1] that phosphorylates Rab10, Rab8a, Rab12 and Rab35 [3]. Overexpression of pathological mutants of LRRK2 that render the kinase hyperactive accounts for 2% of all Parkinson’s cases and up to 20% of the cases in North African Berbers and Ashkenazi Jews [1,14]. Members of the Myosin V family (MYO5A, MYO5B, and MYO5C) are involved in multiple directed vesicle trafficking pathways in epithelial cells [15,16,17]. All three members of the MYO5 family interact with members of the Rab11 family (Rab11a, Rab11b, and Rab25) [13], Rab8a [18], and Rab10 [10]. Rab10 interacts with both MYO5A and MYO5B by binding to the alternatively expressed exon D [10]. The Pfeffer lab discovered a second binding region for Rab10 in the globular tail domain (GTD) of MYO5A that is dependent on the phosphorylation of Rab10 on threonine 73 [11]. Interestingly, here we have shown that pT73-Rab10 does not associate with the GTD of MYO5B. An evaluation of direct interactions using pull-down assays has been difficult because MYO5 interactions with Rab proteins have generally been labile to the solubilization methods necessary for extracting Rab proteins from membranes. Thus, we have mapped the putative pT73-Rab10 binding domain on MYO5A using an assay of colocalization with MYO5A-GTD modifications. Considering the high homology of the MYO5 GTDs, it was surprising that this site was not conserved between the MYO5A and MYO5B GTDs. The three amino acids (MEN, 1473–1475) in MYO5A and corresponding three amino acids in MYO5B (ALA, 1442–1444) regulate the ability of pT73-Rab10 to colocalize with the MYO5A or MYO5B GTDs. In human brain, both the plus exon D and minus exon D forms of MYO5B are found. However, in the brain, only the form lacking exon D and not the exon D-containing form of MYO5A is expressed [10], suggesting that only the phosphorylated form of Rab10 can interact with MYO5A in the brain. MYO5A and another small GTPase, Rac1, control the number of spine-like protrusions found on dendrites [19]. Thus, while many of the functions of MYO5A and MYO5B may be overlapping and complementary, the binding of pT73-Rab10 to the globular tail of MYO5A appears to be specific and likely indicates specific actions of MYO5A at the centrosome.

MYO5A in conjunction with pT73-Rab10 can interact with two members of the Rab Interacting Lysosomal Protein family, RILPL1 and RILPL2 (Rab Interacting Lysosomal Protein Like 1 and 2) [3]. While both RILPL1 and RILPL2 can directly interact with pT73-Rab10 [3], only RILPL2 had been shown to directly interact with MYO5A [19]. Both RILPL1 and RILPL2 are implicated in controlling the trafficking of membrane and signaling proteins to the primary cilia. RILPL1 localizes at the mother centrosome, only coming off during mitosis, while RILPL2 is more transient at the centrosome [20]. Utilizing the overexpression of a pathogenic LRRK2, the Pfeffer lab showed that the phosphorylation status of Rab10 influenced the localization of RILPL2 and MYO5A at the primary cilia [11]. The Pfeffer lab utilized over expression of LRRK2 R1441G in combination with an LRRK2 kinase inhibitor (MLi-2) to alter the ability of RILPL2 and ARL13b to localize to nascent primary cilia. All these studies indicate that MYO5A coordinates activities at the centrosome that do not involve MYO5B.

In summary, the studies presented here indicate that while MYO5A and MYO5B display a number of overlapping molecular associations, MYO5A appears to have a unique site for interaction with phosphorylated Rab10. While we presently have no specific evidence for other interactions of phosphorylated Rab proteins with MYO5 proteins, further investigations are merited to examine such specific protein sequence associations. The conservation of MYO5A and MYO5B interactions with Rab11a may account for tissue-specific compensation by the two motor species mediating common vesicles trafficking pathways in cells that express both motors. The present studies suggest that the interactions of phosphorylated Rab10 with the MYO5A globular tail may regulate more specific influences of MYO5A at the centrosome.

## 5. Conclusions

While MYO5A and MYO5B have many overlapping associations, the interaction of pT3-Rab10 with the globular tail domain is unique to MYO5A. This suggests a specific role for MYO5A in centrosome-related trafficking.

## Figures and Tables

**Table 1:** Cloning and mutagenesis primers.

Primer Name	Primer Sequence
MYO5A-MEN-AAA	ggcgaactagaagtgggccaggcggcggccatatccccaggacagatcat
MYO5B-ALA-MEN	gcccaggacctagaagctgcccagatggagaaccagagtgagaggaagcgccatga
MYO5A-GTD-S-ECOS	gatcgaattccggaaagaaaaggatttccaaggaatgctg

**Table 2:** Antibodies used.

Antibody	Company	Catalog Number	Dilution
Rab10	Cell Signaling (Danvers, MA, USA)	8127P	Western blot: 1:2000 IF: 1:200
pT73-Rab10	Abcam (Waltham, MA, USA)	ab241060	IF: 1:200
pT73-Rab10	Abcam	ab230261	Western blot: 1:1000
Rab11a	Goldenring Lab	VU57	IF 1:400

## Data Availability

Data are contained within the article.

## References

[1] Skelton P.D., Tokars V., Paridiadou L. (2022). LRRK2 at striatal synapses: Cell-type specificity and mechanistic insights. Cells.

[2] Steger M., Tonelli F., Ito G., Davies P., Trost M., Vetter M., Wachter S., Lorentzen E., Duddy G., Wilson S. (2016). Phosphoproteomics reveals that Parkinson’s disease kinase LRRK2 regulates a subset of Rab GTPases. eLife.

[3] Steger M., Diez F., Dhekne H.S., Lis P., Nirujogi R.S., Karayel O., Tonellli F., Martinez T.N., Lorentzen E., Pfeffer S.R. (2017). Systematic proteomic analysis of LRRK2-mediated Rab GTPase phosphorylation establishes a connection to ciliogenesis. eLife.

[4] English A.R., Voeltz G.K. (2013). Rab10 GTPase regulates ER dynamics and morphology. Nat. Cell Biol..

[5] Wauters F., Cornelissen T., Imberechts D., Martin S., Koentjoro B., Sue C., Vangheluwe P., Vandenberghe W. (2020). LRRK2 mutations impair depolarization-induced mitophagy through inhibition of mitochondrial accumulation of RAB10. Autophagy.

[6] Eguchi T., Kuwahara T., Sakurai M., Komori T., Fujimoto T., Ito G., Yoshimura S.-i., Harada A., Fukuda M., Koike M. (2018). LRRK2 and its substrateRab GTPases are sequentially targeted onto stressed lysosomes and maintain their homeostasis. Proc. Natl. Acad. Sci. USA.

[7] Babbey C.M., Bacallao R.L., Dunn K.W. (2010). Rab10 associates with primary cilia and the exocyst complex in renal epithelial cells. Am. J. Physiol. Ren. Physiol..

[8] Ordonez A.J.L., Fernandez B., Fdez E., Romo-Lozano M., Madero-Perez J., Lobbestael E., Baekelandt V., Aiastui A., Munain A.L.d., Melrose H.L. (2019). RAB8, Rab10 and RILPL1 contribute to both LRRK2 kinase-mediated centrosomal cohesion and cilogenesis defictis. Hum. Mol. Genet..

[9] Ordonez A.J.L., Fasiczka R., Fernandez B., Naaldijk Y., Fdez E., Ramirez M.B., Phan S., Boassa D., Hilfiker S. (2022). The LRRK2 signaling network converges on a centriolar phospho-Rab10/RILPL1 complex to cause eficits in centrosome cohesion and cell polarization. Biol. Open.

[10] Roland J.T., Lapierre L.A., Goldenring J.R. (2009). Alternative splicing in class V myosins determines association with Rab10. J. Biol. Chem..

[11] Dhekne H.S., Yanatori I.G., Vides E., Sobu Y., Diez F., Tonelli F., Pfeffer S.R. (2021). LRRK2-phosphorylated Rab10 sequesters Myosin Va with RILPL2 during cilogenesis blockade. Life Sci. Alliance.

[12] Foster K.A., Oster C.G., Mayer M.M., Avery M.L., Audus K.L. (1998). Characterization of the A549 cell line as a type II pulmonary epithelial cell model for drug metabolism. Exp. Cell Res..

[13] Lapierre L.A., Kumar R., Hales C.M., Navarre J., Bhartur S.G., Burnette J.O., Provance J.D.W., Mercer J.A., Bahler M., Goldenring J.R. (2001). Myosin Vb is associated with and regulates plasma membrane recycling systems. Mol. Biol. Cell.

[14] Alessi D.R., Sammler E. (2018). LRRK2 kinase in Parkinson’s disease. Science.

[15] Zhang N., Yao L.L., Li X.D. (2018). Regulation of class V myosin. Cell Mol. Life Sci..

[16] Li J., Lu Q., Zhang M. (2016). Structural Basis of Cargo Recognition by Unconventional Myosins in Cellular Trafficking. Traffic.

[17] Wong S., Weisman L.S. (2021). Roles and regulation of myosin V interaction with cargo. Adv. Biol. Regul..

[18] Roland J.T., Kenworthy A.K., Peranen J., Caplan S., Goldenring J.R. (2007). Myosin Vb interacts with Rab8a on a tubular network containing EHD1 and EHD3. Mol. Biol. Cell.

[19] Lise M.F., Srivastava D.P., Arstikaitis P., Lett R.L., Sheta R., Viswanathan V., Penzes P., O’Connor T.P., El-Husseini A. (2009). Myosin-Va-interacting protein, RILPL2, controls cell shape and neuronal morphogenesis via Rac signaling. J. Cell Sci..

[20] Schaub J.R., Stearns T. (2013). The Rilp-like proteins Rilpl1 and Rilpl2 regulate ciliary membrane content. Mol. Biol. Cell.

